# Peripubertal requirement of Tsg101 in maintaining the integrity of membranous structures in mouse oocytes

**DOI:** 10.1111/cpr.13288

**Published:** 2022-06-29

**Authors:** Hyejin Shin, Dayoung Park, Jiyeon Kim, Min‐Yeong Nam, Sojung Kwon, Da‐Eun Um, Ji‐Eun Oh, Esther Youn, Yhong‐Hee Shim, Kay‐Uwe Wagner, Jin Hyun Jun, Hye‐Ryun Kim, Haengseok Song, Hyunjung Jade Lim

**Affiliations:** ^1^ Department of Veterinary Medicine Konkuk University Seoul Republic of Korea; ^2^ Department of Bioscience and Biotechnology Konkuk University Seoul Republic of Korea; ^3^ Wayne State University School of Medicine and Tumor Biology Program Barbara Ann Karmanos Cancer Institute Detroit Michigan USA; ^4^ Department of Biomedical Laboratory Science Eulji University Seongnam Gyeonggi‐do Republic of Korea; ^5^ Department of Biomedical Science CHA University Seongnam Gyeonggi‐do Republic of Korea; ^6^ Korean Institute of Oriental Medicine Daejeon Republic of Korea; ^7^ Maria S Fertility Hospital Seoul Republic of Korea; ^8^ Maria Plus Fertility Hospital Seoul Republic of Korea; ^9^ Maria Fertility Hospital Seoul Republic of Korea

## Abstract

**Objective:**

As a component of Endosomal Sorting Complex Required for Transport (ESCRT) complex I, the tumor susceptibility gene 101 (Tsg101) carries out multiple functions. In this work, we report that oocyte‐specific deletion of tumor susceptibility gene 101 (Tsg101) leads to age‐dependent oocyte demise in mice.

**Materials and Method:**

*Tsg101* floxed mice (*Tsg101* ^*f/f*^) were bred with *Zp3* ^*cre*^ transgenic mice to examine oocyte‐specific roles of Tsg101. Multiple cellular and molecular biological approaches were taken to examine what leads to oocyte demise in the absence of *Tsg101*.

**Results:**

The death of oocytes from *Zp3* ^*cre*^/*Tsg101* ^*f/f*^ (*Tsg101* ^*d/d*^ thereafter) mice showed a strong correlation with sexual maturation, as gonadotropin‐releasing hormone antagonist injections improved the survival rate of oocytes from 5‐week‐old *Tsg101* ^*d/d*^ mice. Maturation of oocytes from prepubertal *Tsg101* ^*d/d*^ mice proceeded normally, but was largely abnormal in oocytes from peripubertal *Tsg101* ^*d/d*^ mice, showing shrinkage or rupture. Endolysosomal structures in oocytes from peripubertal *Tsg101* ^*d/d*^ mice showed abnormalities, with aberrant patterns of early and late endosomal markers and a high accumulation of lysosomes. Dying oocytes showed plasma membrane blebs and leakage. Blockage of endocytosis in oocytes at 4°C prevented cytoplasmic shrinkage of oocytes from *Tsg101* ^*d/d*^ mice until 9 h. The depletion of *tsg‐101* in *Caenorhabditis elegans* increased the permeability of oocytes and embryos, suggesting a conserved role of *Tsg101* in maintaining membrane integrity.

**Conclusions:**

Collectively, Tsg101 plays a dual role in maintaining the integrity of membranous structures, which is influenced by age in mouse oocytes.

## Graphical abstract

*Tsg101* deficiency in mouse oocytes leads to a complex phenotype involving the plasma membrane (PM). These oocytes are normal until mice reach five weeks of age when oocytes begin to show PM rupture, PM blebbing, and cytoplasmic shrinkage. PM blebbing and cytoplasmic shrinkage can be postponed with a general endocytosis block, but death ensues. In both mouse and in *Caenorhabditis elegans*, *Tsg101* plays a crucial role in maintaining the integrity of PM.

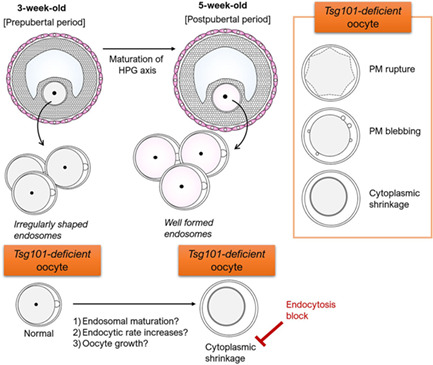

## 1 INTRODUCTION

Maturation of the hypothalamic‐pituitary‐gonadal endocrine axis determines the timing of puberty in mammals.^1^ Gonadotropin‐releasing hormone (GnRH) plays a central role in the sexual maturation process by establishing cyclic rhythms of gonadotropin secretion. Under natural conditions, follicle‐stimulating hormone and luteinizing hormone secretion are regulated by GnRH, and their secretion begins during the peripubertal period.^2^ Overriding such regulation with exogenous gonadotropins, a method widely used in experimental settings, allows immature females to exhibit oocyte maturation and superovulation. The process of oocyte maturation has long been considered a nuclear‐cytoplasmic dichotomy.^3^ Nuclear maturation represents the successful formation of metaphase II chromosomes required for fertilization. Cytoplasmic maturation involves diverse cellular changes that prepare oocytes for activation, fertilization, and subsequent development.^3^ Two‐way interactions between oocytes and somatic cells during follicular development induce various changes that influence oocyte competence.^4^

The endosomal sorting complex required for transport (ESCRT) complexes, ESCRT‐0, ‐I, II, and III, act in a wide array of membrane‐associated events, such as the formation of multivesicular bodies, membrane repair, membrane neck severing during cytokinesis, and exosome formation.^5^ The mammalian ESCRT‐I complex consists of tumor susceptibility gene 101 (Tsg101), vacuolar protein sorting‐associated protein 28 homolog (Vps28), Vps37, and Mvb12.^6^ Tsg101 contains a ubiquitin E2 variant domain at its N‐terminus,^7^ giving the protein the ability to interact with ubiquitin‐tagged proteins for removal via the endolysosomal pathway.^6^ Additionally, Tsg101 acts as an important mediator of midbody formation during cytokinesis via its interaction with Cep55 and ALG2‐interacting protein X (ALIX).^8^ Complete knockout of *Tsg101* causes early embryonic lethality in mice,^9^, ^10^ and depletion of Tsg101 in cells results in impaired endosomal activity, disruption of protein transport through endosomes, and accumulation of multivesicular bodies and lysosomes.^11^, ^12^ Generally, endogenous or overexpressed Tsg101 exhibits puncta‐like patterns in various cell systems.^6^, ^13^ Tsg101 functions with Cep55 and ALIX in cytokinetic events and is localized at the midbody.^14^ During membrane repair, Tsg101 is localized near the plasma membrane (PM).^15^

The PM faces various extracellular insults, and efficient structural repair is crucial for cell survival.^15^ When an injury is introduced to the PM, the ESCRT machinery, particularly ESCRT‐III components and Tsg101, are recruited to the site.^15^ PM damage can be minimized by patching the injury site and shedding wounded PM; here, the ESCRT machinery is involved.^16^ In addition, necroptosis is a newly discovered function of ESCRT components. Tsg101 assists in the translocation of ESCRT‐III components to the cell membrane, delaying membrane permeabilization during necroptotic cell death.^16^ A common feature of PM injury and necroptosis is evident; ESCRT components help to repair damaged PM.

In this study, we explored the consequence of *Tsg101* deletion in mouse oocytes by crossing *Tsg101* floxed mice with *Zp3* ^*cre*^ transgenic mice.^10^, ^17^ The results indicate that Tsg101 is involved in maintaining the integrity of the PM and in endolysosomal maturation in oocytes in an age‐dependent manner. This phenotype provides insights into the potential mechanism by which cellular changes involving Tsg101 occur during the peripubertal period to produce competent oocytes.

## 2 MATERIALS AND METHODS

### 2.1 Mouse breeding and genotyping

All mice were maintained in accordance with the policies of the Konkuk University Institutional Animal Care and Use Committee (approval numbers KU17067, KU18106, KU19078, KU20036, and KU21035). *Zp3* ^*cre*^ transgenic mice [C57BL/6‐Tg(Zp3‐cre)93Knw/J] were obtained from The Jackson Laboratory (Bar Harbor, ME, USA).^17^ *Tsg101* floxed mice (*Tsg101* ^*f/f*^)^10^ were bred with *Zp3* ^*cre*^ mice with a 129/SvJ genetic background. Genomic DNA was extracted from mouse tails for genotyping. PCR was performed using the primers listed in Table S1.

### 2.2 Isolation and culture of mouse oocytes

**FIGURE 1:**
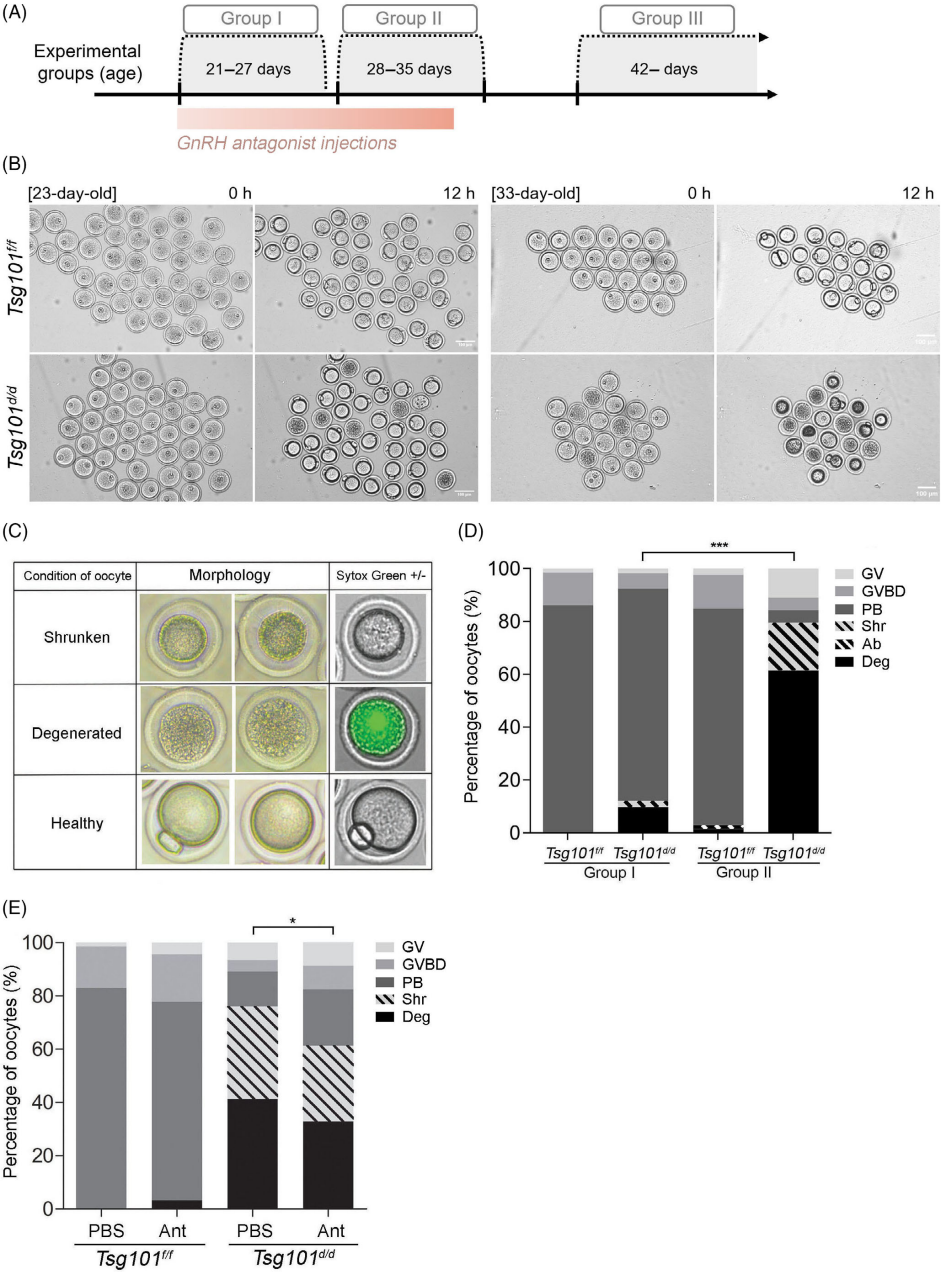
Oocytes from *Tsg101* ^*d/d*^ mice exhibit an age‐dependent death phenotype. (A) Experimental groups used in this study. Group I, prepubertal mice sacrificed between 21 and 27 days of age; Group II, peripubertal mice sacrificed between 28 and 35 days of age; and Group III, mice sacrificed after 42 days of age. (B) *In vitro* maturation of oocytes from *Tsg101* ^*f/f*^ and *Tsg101* ^*d/d*^ mice at 23 and 33 days of age. The images were obtained by JuLi^TM^ time‐lapse microscopy at 1‐h intervals for 12 h in culture, and representative images at the indicated hours are shown (see Movies [Link], [Link]). (C) Criteria for determining the morphological characteristics of oocytes from *Tsg101* ^*d/d*^ mice in various experiments. (D) *In vitro* maturation rates of oocytes from Groups I and II *Tsg101* ^*f/f*^ and *Tsg101* ^*d/d*^ mice. The detailed number of oocytes in each group is presented in Table 1. Because the experiments were always terminated after 12 h in culture, a small percentage of *Tsg101* ^*f/f*^ oocytes was not fully mature. Statistical significance was determined using the chi‐square test between Deg. versus others (GV + GVBD + PB + shrunken): ***p < 0.0001. Ab, abnormal 2‐cell‐like morphology; Deg., degenerated; GV, germinal vesicle; GVBD, germinal vesicle breakdown; PB, polar body. (E) *Tsg101* ^*f/f*^ and *Tsg101* ^*d/d*^ mice (21 days old) received daily i.p. injections of a GnRH antagonist (ganirelix acetate, Ant) for 13 days, from day 21 to 33. the average number of ovulations increased from 7.7 to 13.7 after GnRH antagonist injection in *Tsg101* ^*d/d*^ mice. Statistical significance was determined using the chi‐square test between Deg. + Shrunken versus GV + GVBD + PB: *p < 0.05.

Oocytes from *Tsg101* ^*d/d*^ mice showed differing rates of shrinkage and death; thus, they were grouped into Groups I, II, and III depending on the age of the female mice (Figure 1A). The collection of germinal vesicle (GV) and polar body PM oocytes as described elsewhere.^18^ Briefly, mice received an intraperitoneal (i.p.) injection of 5 IU pregnant mare serum gonadotropin (PMSG; Sigma‐Aldrich, St. Louis, MO, USA). Fully grown cumulus‐oocyte complexes (COC) were collected by ovary puncture. The cumulus cells were removed by repetitive pipetting using a glass capillary. For *in vitro* maturation experiments, GV‐stage oocytes were cultured *in vitro* for 12–24 h in an M16 medium (M7292; Sigma‐Aldrich) under mineral oil (CooperSurgical, Inc., Trumbell, CT, USA) at 37°C in a humidified atmosphere containing 5% CO_2_. The status of oocytes was scored at the indicated time points. For the collection of ovulated COC, mice received 5 IU of human chorionic gonadotropin (hCG) at 48 h after PMSG injection. Ovulated COCs were retrieved from the oviducts at 13 h after hCG administration, and treated with hyaluronidase to remove cumulus cells. In some experiments, oocyte maturation was recorded from the GV stage for 12 h using a JuLI^TM^ time‐lapse microscope (NanoEnTek, Seoul, Korea). Images were automatically taken at 1‐h intervals and converted into a video file (Movies [Link], [Link]).

### 2.3 Embryo collection and culture

Embryos were collected from Group I or Group II *Tsg101* ^*f/f*^ and *Tsg101* ^*d/d*^ mice that were bred with stud Institute of Cancer Research (ICR) male mice. Female mice were induced to superovulate with PMSG (2.5 IU) and hCG injections and caged with sexually mature male mice immediately after the administration of hCG. At 40–41 h after the administration of hCG, oviducts were collected for oviductal flushing. Retrieved zygotes were transferred to Potassium‐supplemented simplex optimised medium (KSOM) media (Sigma‐Aldrich) and cultured at 37°C in 5% CO_2_.

### 2.4 Intraperitoneal injection of GnRH antagonist

*Tsg101* ^*f/f*^ and *Tsg101* ^*d/d*^ mice (21 days old) received daily i.p. injections of ganirelix acetate solution in Phosphate buffered saline (PBS) (LG Chemicals, Seoul, Korea) at 0.5 mg/kg body weight for 13 days. On the 11th day after injection, the mice also received PMSG injection. The mice were sacrificed at the age of 34 days, and GV oocytes were collected and observed under JuLI for 12 h. Five sets of experiments were performed using 8 (135 oocytes in PBS group) and 6 (90 oocytes in GnRH antagonist group) *Tsg101* ^*f/f*^ mice and 12 (92 oocytes in the PBS group) and 10 (137 oocytes in the GnRH antagonist group) *Tsg101* ^*d/d*^ mice.

### 2.5 Ovarian cryosections

Ovaries were removed from *Tsg101* ^*f/f*^ and *Tsg101* ^*d/d*^ mice at the indicated ages and fixed in 4% paraformaldehyde in PBS at 4°C overnight. Ovaries were embedded in 30% sucrose in PBS for 4 h at 4°C, mounted with optimal cutting temperature compound (Polysciences, Inc., Warrington, PA, USA), and snap‐frozen in liquid nitrogen. Next, the optimal cutting temperature‐embedded frozen sections (12‐μm thickness) were subjected to immunofluorescence staining.

### 2.6 Immunofluorescence staining

Immunofluorescence staining of cryosections or oocytes was performed as described previously.^19^ Briefly, sections or oocytes were fixed in 4% paraformaldehyde in PBS for 20 min and washed with PBS. They were then permeabilized with 0.1% Tween‐20 in PBS for 20 min and washed with PBS. The specimens were blocked with 2% bovine serum albumin (BSA) in PBS for 1 h at 25°C, followed by incubation with indicated primary antibodies in 2% BSA/PBS for 2 h at 25°C. After washing in 2% BSA/PBS for 5 min, the sections were incubated with Alexa Fluor 488‐conjugated secondary antibody (1:250; Thermo Fisher Scientific, Waltham, MA, USA) in 2% BSA/PBS for 40 min. The sections were washed and counterstained with TOPRO^TM^‐3‐iodide (1:100; T3605; Thermo Fisher Scientific).

The primary antibodies used were anti‐Histone H3 (rabbit polyclonal, ab183626; Abcam, Cambridge, UK), anti‐Cep55 (rabbit polyclonal, ab170414; Abcam, Cambridge, UK), anti‐mouse vasa homolog (MVH) (rabbit polyclonal, ab13840; Abcam, Cambridge, UK), anti‐EEA‐1 (rabbit polyclonal, 2411S; Cell Signaling Technologies, Danvers, MA), anti‐Rab7 (rabbit monoclonal, 9367; Cell Signaling Technologies), and anti‐Lamp‐1 (rat monoclonal, NB100‐77683; Novus Biologicals, Littleton, CO, USA). Images were obtained using a Zeiss LMS900 confocal microscope (Oberkochen, Germany) and analyzed using the ZEN software (Zeiss).

### 2.7 Confocal live imaging

Oocytes were stained with CellMask^TM^ Plasma Membrane Stain (2.5 μg/ml; C10046; Life Technologies, Carlsbad, CA, USA), Sytox^TM^ Green Nucleic Acid Stain (2.5 μM; S7020; Invitrogen, Waltham, MA), or ER‐Tracker^TM^ Red dye (1:1000; E34250; Thermo Fisher). The oocytes were washed three times in an M16 medium and observed by confocal live imaging using a Zeiss LMS900 confocal microscope.

### 2.8 RNA extraction and quantitative PCR analysis

GV or PB oocytes were collected from several ICR female mice at the indicated ages. RNA extraction and quantitative PCR analyses were performed following the protocol previously described.^18^ Relative gene expression was normalized to that of histone H2A.z (*H2afz*) mRNA expression,^20^ and relative quantification was calculated using the 2^−ΔΔCt^ method.^21^ All experiments were conducted using three independent sets of samples and performed in duplicate.

### 2.9 Oocyte maturation at 4°C

GV oocytes were obtained from several Group II *Tsg101* ^*f/f*^ and *Tsg101* ^*d/d*^ mice, randomly grouped, and were cultured *in vitro* for 24 h in M2 media (M7167, Sigma‐Aldrich) under mineral oil at 4°C in a refrigerator or 37°C incubators. In some experiments, oocytes in a 37°C incubator were treated with 3‐Isobutyl‐l‐methyl xanthine (IBMX, #I5879; Sigma‐Aldrich)^22^ at 200 μM for 12–20 h.

### 2.10 Transmission electron microscopy

GV oocytes from *Tsg101* ^*f/f*^ and *Tsg101* ^*d/d*^ mice were fixed with 2.5% glutaraldehyde (Sigma‐Aldrich) in PBS (pH 7.2) for 2 h at 25°C and then washed with PBS. Oocytes were then placed in agar chips for pre‐embedding and post‐fixed in 1% osmium tetroxide (Sigma‐Aldrich) in PBS. Chips with oocytes were dehydrated and infiltrated with Epon 812 (Sigma‐Aldrich). Semi‐thin sections were prepared using an Ultracut Ultramicrotome equipped with a diamond knife (Leica, Wetzlar, Germany) and placed on copper grids. The sections were double‐stained with 2% uranyl acetate and lead citrate and examined under an H7600 transmission electron microscope (80 kV; Hitachi, Tokyo, Japan).

### 2.11 *In vitro* transcription and microinjection of EGFP‐Tsg101

To visualize Tsg101 localization in mouse oocytes, GFP‐tagged full‐length *Tsg101* DNA constructs were transcribed *in vitro*. *Enhanced green fluorescent protein* (*EGFP*)‐*Tsg101* cRNA was microinjected into GV oocytes from 4‐week‐old ICR mice at a concentration of 1 μg/μl in the presence of IBMX, followed by incubation for 8 h to allow gene expression. The oocytes were then transferred to IBMX‐free M16 medium and cultured for 12 h. The injection volume was 5–10 pL per oocyte, which is approximately 1%–3% of the total oocyte volume. During injection and recovery after injection, the oocytes were placed in droplets of M16 medium with 200 μM IBMX for 12 h. Microinjection was performed using a micromanipulator (Narishige, Tokyo, Japan) with a picoinjector (Femto Jet 4i; Eppendorf, Hambrug, Germany).

### 2.12 *Caenorhabditis elegans* and maintenance

The *Caenorhabditis elegans* strain *DCL569: mkcSi13 [sun‐1p::rde‐1::sun‐1 3'UTR + unc‐119(+)] II; rde‐1(mkc36) V* was used to observe the effects of germline‐specific RNAi‐mediated depletion of *tsg‐101*. The animals were incubated and experimented with at 20°C on nematode growth medium agar plates seeded with *Escherichia coli* strain OP50, as previously described.^23^

### 2.13 Soaking RNA interference

RNA interference (RNAi) was performed using soaking methods, as previously described.^24^ The cDNA template of *tsg‐101*, flanked by T7 promoter sequences in pL4440, was amplified by PCR using the T7 primer, 5′‐GTAATACGACTCACTATAGGGC‐3′, and L4440 T7 primer, 5′‐ATTAATACGACTCACTATAGGGA‐3′. To synthesize dsRNA of *tsg‐101*, *in vitro* transcription was performed using a cDNA template. For the mock RNAi control, a soaking buffer was used without dsRNA. Synchronized L4‐stage animals were soaked in *tsg‐101* dsRNA solution for 24 h at 20°C, incubated in fresh nematode growth medium agar plates containing OP50 for 24 or 48 h, and examined. Each animal incubated for 24 and 48 h after recovery from the dsRNA solution was defined as a 1‐day adult and 2‐day adult, respectively.

### 2.14 Fertility assessment in *C. elegans*

To assess animal fertility, the number of progeny and embryonic lethality was scored as previously described.^24^ Each 2‐day adult was transferred to a new nematode growth medium plate and incubated for 24 h at 20°C. After removing the mother, the number of laid eggs and hatched larvae were counted as the total number of progenies. The progenies were incubated for an additional 24 h at 20°C, and non‐hatched eggs on the plates were considered dead. Embryonic lethality was calculated by dividing the number of non‐hatched eggs by the total number of progenies.

### 2.15 Permeability analysis in *C. elegans*

To analyze the membrane permeability of oocytes and embryos, they were stained with FM4‐64 dye (Sigma‐Aldrich), as previously described.^24^ The 1‐day adult and 2‐day adult were dissected to extrude gonads and embryos in 150 mM KCl containing FM4‐64 dye (30 μM). The penetration of FM4‐64 dye into oocytes and embryos was observed using a microscope (Zeiss).

### 2.16 Statistical analysis

Data analysis and graphing were performed using GraphPad Prism 5 (GraphPad, Inc., La Jolla, CA, USA). Statistical significance was assessed by chi‐squared test, two‐way analysis of variance, or Student's *t*‐test. We performed the Shapiro‐Wilk test before using a parametric analysis. The analysis method is shown in the figure legend. Statistical significance is marked as follows: *p < 0.05, **p < 0.01, and ***p < 0.001.

## 3 RESULTS

### 3.1 Oocytes from peripubertal *Tsg101* ^*d/d*^ mice exhibit dual abnormalities

*Tsg101* is expressed in mouse oocytes, cumulus cells, and the whole ovary (Figure S1A). Deletion of floxed *Tsg101* by the oocyte‐specific *Zp3* ^*cre*^ promoter‐driven Cre was confirmed in oocytes from 4‐week‐old *Zp3* ^*cre*^/*Tsg101* ^*f/f*^ (*Tsg101* ^*d/d*^ thereafter) mice (Figure S1A). We first examined whether oocytes from *Tsg101* ^*d/d*^ mice matured normally. GV (prophase I) stage oocytes were collected from *Tsg101* ^*f/f*^ and *Tsg101* ^*d/d*^ mice and matured *in vitro*. During this initial survey, we noted that the phenotype clearly differed between prepubertal mice aged 22–27 days (Group I) and peripubertal mice aged older than 28 days (Group II, Figure 1A,B). We also observed two different patterns of anomalies in oocytes from *Tsg101* ^*d/d*^ mice (Figure 1C). “Shrunken” oocytes show cytoplasmic shrinkage but have intact PM (Sytox Green‐negative). “Degenerated” oocytes show flattened cytoplasm and are Sytox Green‐positive, suggesting leaky PM (Figure 1C). Oocytes from Group I and II mice were scored according to these criteria (Figure 1C). As shown in Figure 1D, approximately 10% of oocytes from Group I *Tsg101* ^*d/d*^ mice showed abnormalities, whereas up to 80% of oocytes from Group II *Tsg101* ^*d/d*^ mice were either degenerated or shrunken by 12 h (Table 1, hormone‐primed). Time‐lapse microscopy images (Movies [Link], [Link]) revealed that GV oocytes from Group II *Tsg101* ^*d/d*^ mice showed cytoplasmic shrinkage and rupture within the first several hours of culture (Movie S4). As these experiments were performed under gonadotropin treatment, we also examined oocyte maturation in random cycling 34‐day‐old mice (Group II) to rule out any influence of exogenous gonadotropins. Similar results with a high rate of abnormalities were observed in oocytes from Group II *Tsg101* ^*d/d*^ mice (Table 1, random cycling).

**TABLE 1:** *In vitro* maturation of oocytes from *Tsg101* ^*d/d*^ mice

Hormone‐primed (Figure 1D)^a^										
Group	Genotype	No. of mice	Total GV	Average GV	12 h later					
					GV (%)	GVBD (%)	PB (%)	Ab (%)	Shr. (%)	Deg. (%)
I	*Tsg101* ^*f/f*^	7	138	19	2 (1.45)	17 (12.32)	119 (86.23)	0	0	0
	*Tsg101* ^*d/d*^	8	173	21	3 (1.73)	10 (5.78)	139 (80.35)	0	4 (2.31)	17 (9.83)
II	*Tsg101* ^*f/f*^	10	132	13	3 (2.27)	17 (12.88)	108 (81.82)	2 (1.52)	0	2 (1.52)
	*Tsg101* ^*d/d*^	14	83	6	9 (10.84)	4 (8.82)	4 (4.82)	0	15 (18.07)	51 (61.45)

Random cycling^b^									
34‐day‐old				>21 h later					
Genotype	No. of mice	Total GV	Average GV	GV	GVBD	PB	Ab.	Shr.	Deg.
*Tsg101* ^*f/f*^	4	45	11.3	0	3	41	1	0	0
*Tsg101* ^*d/d*^	3	29	9.7	0	2	8	2	3	14

Abbreviations: Ab, abnormal 2‐cell‐like morphology; Deg., degenerated; GV, germinal vesicle; GVBD, germinal vesicle breakdown; PB, polar body; Shr, shrunken ooplasm.

^a^ Mice were sacrificed at 48 h post‐PMSG injection and GV oocytes were collected from fully grown cumulus‐oocyte complexes. They were cultured *in vitro* for 12 h and then scored for the maturation stages.

^b^ Random cycling mice were sacrificed and ovaries were punctured. COCs were collected from large follicles. They were cultured *in vitro* for up to 24 h and then scored for the maturation stages.

Next, we examined the status of ovulated oocytes (with a polar body, PB oocytes) from *Tsg101* ^*d/d*^ mice. In Group II *Tsg101* ^*d/d*^ mice, the number of PB oocytes was low (average 6.8 vs. 18 in the control group), and most ovulated oocytes were degenerated (Figure S1B). The phenotype was more severe in Group III *Tsg101* ^*d/d*^ mice (Figure 1C). The nuclear configuration at each stage of oocyte maturation was examined by immunofluorescence staining for histone H3 and Cep55 (Figure S2) in surviving oocytes from Group II *Tsg101* ^*d/d*^ mice, and it appeared normal. Thus, oocyte abnormalities in *Tsg101* ^*d/d*^ mice were similarly observed both *in vitro* and in vivo and were clearly age‐dependent. As *Tsg101* ^*d/d*^ mice age, the number of surviving oocytes decreases and degenerated oocytes in numerous states was observed along with them.

The status of the primordial germ cell pool in different age groups (age 8, 30, and 54 days) was examined by immunofluorescence staining of MVH, a marker of primordial germ cells.^25^ As shown in Figure S3, the status of the MVH‐positive primordial germ cell population near the ovarian surface was comparable between the age‐matched groups of *Tsg101* ^*f/f*^ and *Tsg101* ^*d/d*^ ovaries, showing that germ cell depletion is not observed in *Tsg101*‐deficient oocytes. Considering that the *Zp3* ^*cre*^ promoter deletes the targeted floxed gene as early as postnatal day 5,^26^ the age‐distinct phenotype of oocytes in *Tsg101* ^*d/d*^ mice is fully exhibited when ovulation is imminent. In naturally cycling 12‐week‐old mice, *Tsg101*‐deficient oocytes within some of the largest follicles exhibited deformities (Figure S4, arrows), whereas oocytes within early follicles (primordial, primary, and secondary follicles) showed normal morphology, as in *Tsg101* ^*f/f*^ mice.

### 3.2 Preimplantation embryo development proceeds normally in prepubertal *Tsg101* ^*d/d*^ mice

The developmental competence of surviving oocytes from *Tsg101* ^*f/f*^ and *Tsg101* ^*d/d*^ mice was assessed. Fertilization and embryonic development rates of *Tsg101* ^*d/d*^ mice on day 2 of pregnancy were examined. As shown in Table 2, oocytes from Group I *Tsg101* ^*d/d*^ mice were fertilized and proceeded normally to the blastocyst stage. However, most oocytes from Group II *Tsg101* ^*d/d*^ mice were already in a state of death at the beginning of the culture (47 of 64 oocytes), and the surviving oocytes were unfertilized (13 of 17 surviving oocytes, Table 2). The results are summarized as follows: oocytes from Group I *Tsg101* ^*d/d*^ mice fully exhibited developmental competence, but Group II mice exhibited oocyte anomalies that precluded them from producing competent preimplantation embryos.

**TABLE 2:** Preimplantation embryonic development in *Tsg101* ^*d/d*^ mice

Day 2	Genotype	No. of mice	Total ovulations	Average ovulations	Unfertilized (%)	2‐cell embryo (%)	Degenerated (%)
Group I	*Tsg101* ^*f/f*^	2	61	30	4 (6.5)	57 (93.5)	0 (0)
	*Tsg101* ^*d/d*^	6	89	15	9 (10.1)	79 (88.8)	1 (1.1)
Group II	*Tsg101* ^*f/f*^	7	161	23	14 (8.70)	115 (71.43)	32 (19.87)
	*Tsg101* ^*d/d*^	9	64	7	13 (20.31)	4 (6.25)	47 (73.44)

Day 5	Genotype	No. of mice	Blastocyst^a^ (%)	Morula (%)	2‐ to 4‐cell (%)	Unfertilized (%)	Degenerated (%)
Group I	*Tsg101* ^*f/f*^	2	46 (75.4)	9 (14.8)	0 (0)	2 (3.3)	0 (0)
	*Tsg101* ^*d/d*^	6	74 (82.2)	2 (2.2)	3 (3.4)	1 (1.1)	6 (7.7)
Group II	*Tsg101* ^*f/f*^	7	109 (67.7)	0 (0)	0 (0)	1 (0.62)	0 (0)
	*Tsg101* ^*d/d*^	9	4 (6.25)	0 (0)	0 (0)	1 (1.56)	1 (1.56)

*Note*: Two‐cell stage embryos were collected on day 2 of pregnancy and cultured for 4 days in KSOM media. Developmental rates of embryos obtained from day 2 pregnant mice (at the beginning of culture) and day 5 (72 h post‐culture) are shown.

^a^ Includes early and expanded blastocysts.

### 3.3 Antagonizing GnRH secretion alleviates the death phenotype of oocytes from *Tsg101* ^*d/d*^ mice

The peripubertal transition of the phenotype in oocytes from *Tsg101* ^*d/d*^ mice suggests that GnRH‐activated onset of puberty and subsequent physiological changes exert external influences on *Tsg101* ^*d/d*^ mice. To delay sexual maturation, we injected a GnRH antagonist into *Tsg101* ^*f/f*^ and *Tsg101* ^*d/d*^ mice daily for 13 days from day 21,^27^ and oocytes were examined when the mice were 34 days old. GnRH antagonist treatment of *Tsg101* ^*d/d*^ mice not only increased the number of ovulations (from 7.7 oocytes in PBS‐injected *Tsg101* ^*d/d*^ mice vs. 13.7 oocytes in Ant‐injected *Tsg101* ^*d/d*^ mice) but also reduced shrinkage and death rates of oocytes (Figure 1E). The results show that the peripubertal transition of the oocyte phenotype in *Tsg101* ^*d/d*^ mice is influenced by GnRH‐induced sexual maturation.

### 3.4 Membrane bleb formation in oocytes from Group II *Tsg101* ^*d/d*^ mice

**FIGURE 2:**
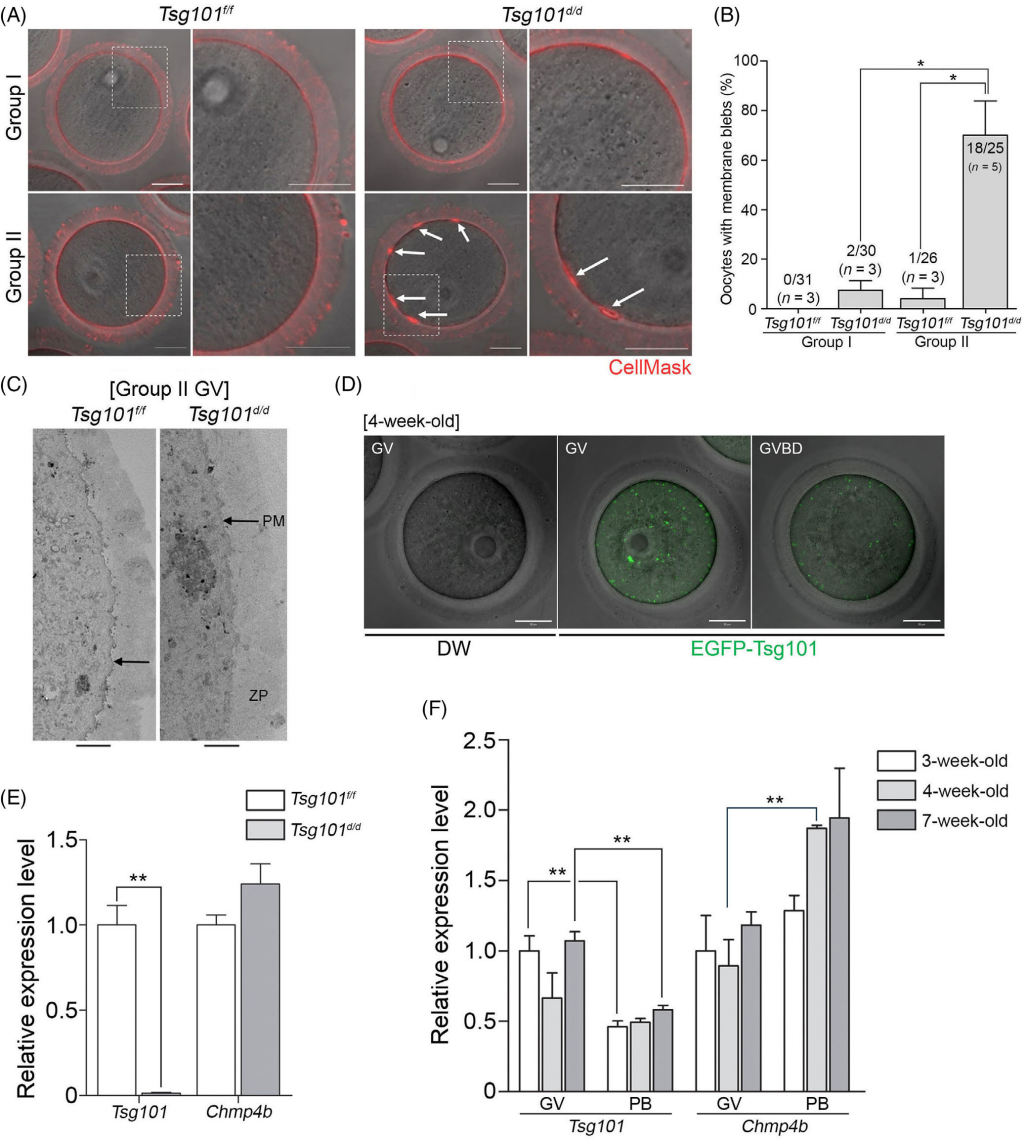
Properties of cell death in oocytes from *Tsg101* ^*d/d*^ mice. (A) GVs were stained with CellMask^TM^ Deep Red Membrane Stain (2 μg/ml, red) in M16 medium. The boxed areas are enlarged in panels on the right. White arrows indicate membrane blebs in the perivitelline space of *Tsg101* ^*d/d*^ oocytes. Scale bar = 20 μm. (B) The number of oocytes with membrane blebs was counted. The number of bleb‐positive oocytes per total number of oocytes is indicated at the top of each bar. Values represent mean ± SEM. One‐tailed *t*‐test. *p < 0.05. (C) Transmission electron microscopy images of the peripheries of oocytes from Group II *Tsg101* ^*f/f*^ and *Tsg101* ^*d/d*^ mice. PM, plasma membrane; ZP, zona pellucida. (D) Tsg101 localization was assessed by microinjection of EGFP‐tagged *Tsg101* cRNA. Scale bar = 20 μm. Note green puncta‐like signal of EGFP‐Tsg101. (E) Quantitative PCR (qPCR) analysis of *Tsg101* and *Chmp4b* in *Tsg101* ^*f/f*^ and *Tsg101* ^*d/d*^ levels in PB oocytes from 4‐week‐old mice. Two‐tailed *t*‐test. **p < 0.01. Values represent mean ± SEM. (F) qPCR analysis of *Tsg101* and *Chmp4b* in GV and PB oocytes from 3‐, 4‐, and 7‐week‐old ICR mice. Two‐tailed *t*‐test. **p < 0.01. Values represent mean ± SEM.

Tsg101 and ESCRT‐III components were shown to be required for the repair of PM lesions.^15^, ^16^ The death of oocytes from Group II *Tsg101* ^*d/d*^ mice was accompanied by an apparent PM rupture (Movie S4). Thus, we assessed the status of the PM using live confocal imaging. CellMask^TM^ deep red live imaging^28^ revealed uniform patterns of ring‐shaped PM peripheries in most oocytes from *Tsg101* ^*f/f*^ and Group I *Tsg101* ^*d/d*^ mice, whereas approximately 70% of oocytes from Group II *Tsg101* ^*d/d*^ mice showed conspicuous membrane blebs in the perivitelline space (Figure 2A,B). Membrane integrity in *Tsg101* ^*d/d*^ oocytes appeared to be compromized at the ultrastructural level, as the PM under the zona pellucida in *Tsg101* ^*d/d*^ oocytes appeared fuzzy and blurred compared with the clear boundary in *Tsg101* ^*f/f*^ oocytes (Figure 2C). These data revealed physical defects in the PM of Group II *Tsg101* ^*d/d*^ oocytes.

As the localization of ESCRT components in oocytes is unknown, we examined Tsg101 localization by microinjecting *EGFP‐Tsg101* complementary RNA (cRNA) into wild‐type GV oocytes. The EGFP‐Tsg101 construct showed puncta‐like patterns when expressed in NIH3T3 cells (Figure S5A). At the GV stage, EGFP‐Tsg101 exhibited puncta‐like patterns in oocytes (Figure 2D). The signal was typically weak and rapidly decreased as oocytes matured to the PB stage (Figure S5B). Notably, we observed that the EGFP‐Tsg101 signal was heavily concentrated near the PM of a microinjected degenerating oocyte (Figure S5B, right panels). This pattern may be analogous to the previously reported localization of Tsg101 at the site of membrane damage.^15^

We confirmed that there was no remnant *Tsg101* mRNA in oocytes from *Tsg101* ^*d/d*^ mice (Figure 2E). To determine whether there was an age‐dependent expression of Tsg101 in mouse oocytes, qPCR was performed using oocytes from 3‐, 4‐, and 7‐week‐old mice. There was no significant difference in the expression of *Tsg101* or *Chmp4b* between the different age groups (Figure 2F). Notably, a reduction in *Tsg101* expression was noted in polar body (PB) oocytes compared with GV oocytes (Figure 2F).

### 3.5 Age‐dependent progressive deterioration of endolysosomal structure in oocytes of *Tsg101* ^*d/d*^ mice

**FIGURE 3:**
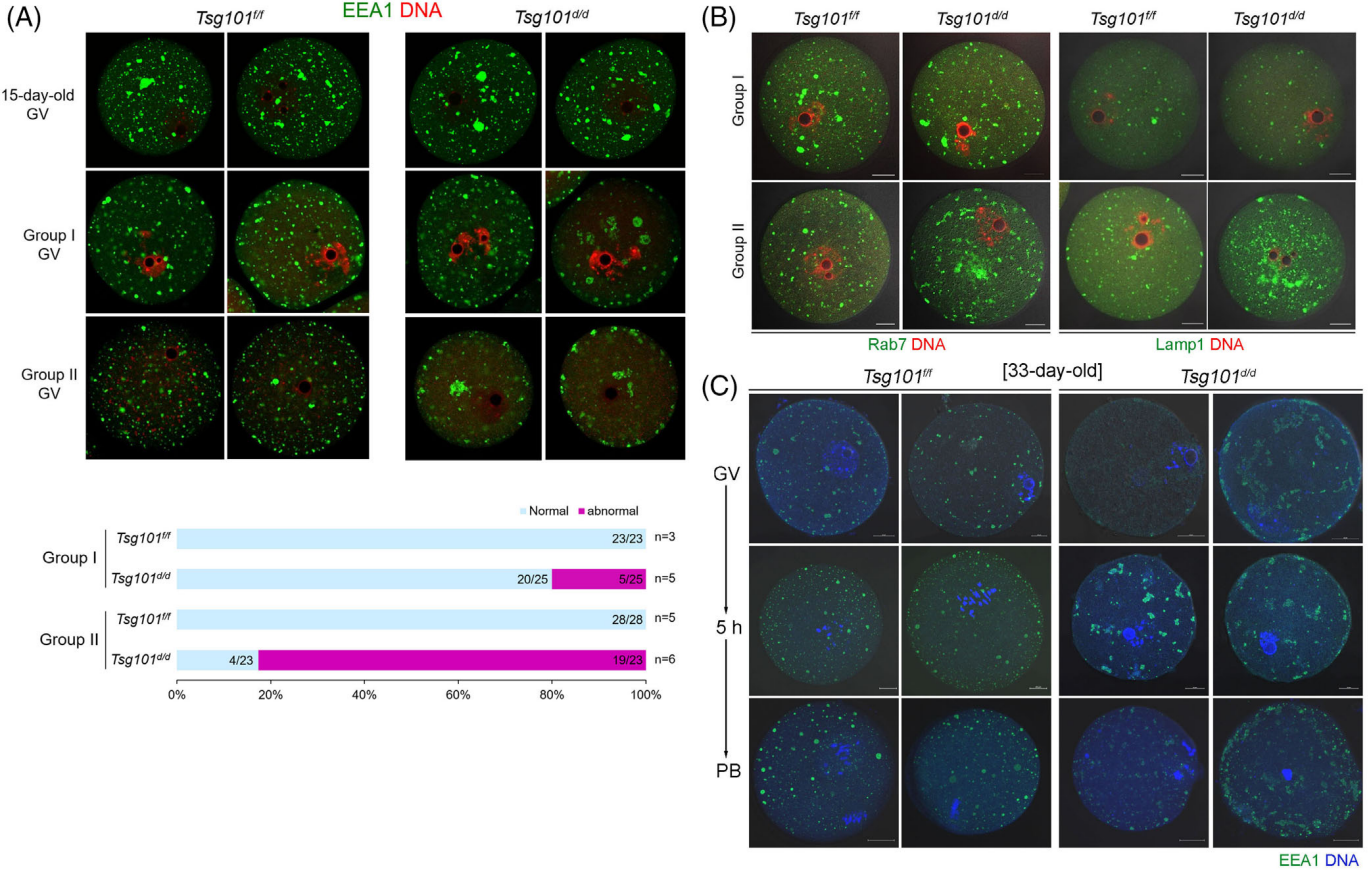
Endolysosomal abnormalities in oocytes from *Tsg101* ^*d/d*^ mice are age‐dependent. (A) GV oocytes from *Tsg101* ^*f/f*^ and *Tsg101* ^*d/d*^ mice at the indicated ages were stained with anti‐EEA1 antibody. The graph shows oocytes with an abnormal EEA1 pattern. The numbers of oocytes are shown within bars, and the numbers of mice are given at the end of each bar. (B) GV oocytes were stained with anti‐Rab7a (late endosome marker) and anti‐Lamp‐1 (lysosome‐specific marker) antibodies. Scale bar = 20 μm. (C) Pattern of EEA‐1 localization in oocytes during maturation.

The function of Tsg101 as a component of ESCRT I centers on endolysosomal systems, such as endosomal trafficking and maturation.^11^ Early endosome antigen 1 (EEA1, an early endosome marker), Ras‐related protein 7a (Rab7a, a late endosome marker), and lysosomal‐associated membrane protein 1 (Lamp1, a lysosome marker) were used to monitor distinct stages of endolysosomal maturation in mouse oocytes. In immature (15‐day‐old) mice, EEA1 puncta in oocytes were more irregular in shape than those in oocytes of Group I and II (Figure 3A). At this age, there was no difference in the pattern between oocytes from the control and *Tsg101* ^*d/d*^ mice. EEA1‐positive puncta became rounder and smaller in oocytes from older mice. The EEA1 pattern of oocytes from Group II *Tsg101* ^*d/d*^ mice widely deviated from a punctate pattern; large EEA1 aggregates were observed near the nucleus and at the periphery, and many showed malformed tubular patterns (Figure 3A). The number of oocytes with abnormalities increased in Group II (Figure 3A, graph). Rab7a and Lamp1 also showed abnormal patterns in *Tsg101* ^*d/d*^ oocytes, with a profound increase in Lamp1 signal (Figure 3B). Thus, *Tsg101* deletion in oocytes leads to abnormal endolysosomal structures, but not before sexual maturation.

We also examined the EEA1 distribution pattern during oocyte maturation (Figure 3C). During the maturation of oocytes from Group II *Tsg101* ^*f/f*^ mice, EEA1‐positive puncta became rounder and larger and appeared to be more localized in the oocyte periphery. In oocytes from *Tsg101* ^*d/d*^ mice, these changes were indiscernible because the EEA1 pattern was abnormal from the GV stage. As the endoplasmic reticulum is associated with the dynamic positioning of endolysosomal structures,^29^ we examined the distribution of the endoplasmic reticulum in these oocytes (Figure S6). The endoplasmic reticulum pattern was similar in all groups of oocytes from *Tsg101* ^*f/f*^ and *Tsg101* ^*d/d*^ mice. These results suggest that endolysosomal structures in oocytes develop dynamically as mice age and oocytes mature.

### 3.6 Blockage of endocytosis at low temperature reduces shrinkage of oocytes from Group II *Tsg101* ^*d/d*^ mice

**FIGURE 4:**
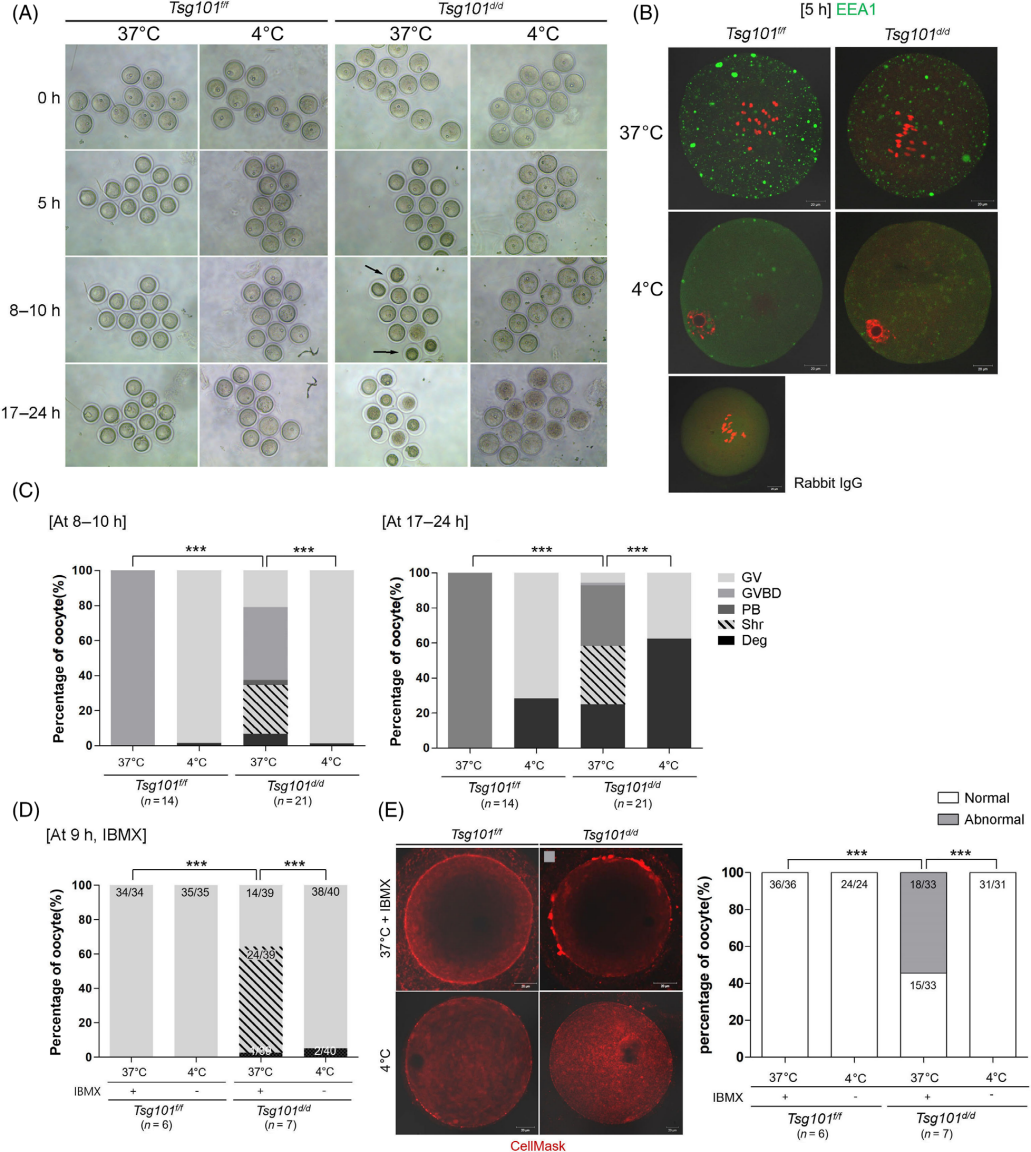
Blockage of endocytosis in cold temperature reduces shrinkage rate and PM bleb formation in oocytes from Group II *Tsg101* ^*d/d*^ mice. (A) Effect of blocking endocytosis during oocyte maturation. GV oocytes were collected from Group II *Tsg101* ^*f/f*^ and *Tsg101* ^*d/d*^ mice, cultured in M2 medium at 37 or 4°C, and observed for 24 h. At 8–10 h, the number of oocytes from *Tsg101* ^*d/d*^ mice with shrunken cytoplasm (black arrows) was noticeably lower at 4°C than at 37°C. (B) Immunofluorescence staining of anti‐EEA1 in oocytes at 5 h at 4°C. Scale bar = 20 μm. (C) Maturation and survival rates of oocytes from *Tsg101* ^*f/f*^ and *Tsg101* ^*d/d*^ mice at 37 or 4°C (Table S2). Statistical significance was measured by the chi‐squared test (two‐sided) between shrunken and others (GV + GVBD + PB + Deg.): ***p < 0.0001. (D) GV oocytes from *Tsg101* ^*f/f*^ and *Tsg101* ^*d/d*^ mice were cultured for 9 h in M2 with IBMX (200 μM) at 37°C to prevent GVBD, or in M2 without IBMX at 4°C. This was to create a similar condition of blocked GVBD at both 37 and 4°C. The graph shows the maturation and survival rates of oocytes from *Tsg101* ^*f/f*^ and *Tsg101* ^*d/d*^ mice at the indicated temperatures. The graph legend is shown in (C). The number of oocytes per total number of oocytes is shown in each bar. Statistical significance was measured by the chi‐squared test (two‐sided) between shrunken and others (GV + GVBD + PB + Deg.). ***p < 0.0001. (E) Comparison of PM bleb formation in oocytes from *Tsg101* ^*f/f*^ and *Tsg101* ^*d/d*^ mice cultured at 4°C. Oocytes were stained with CellMask^TM^ deep red membrane (red). “Abnormal” indicates oocytes with PM blebs (see the photomicrograph with gray rectangle). The number of oocytes per total sample is shown for each bar. Statistical significance was measured using the chi‐square test (two‐sided) between normal and abnormal oocytes. ***p < 0.0001. Scale bar = 20 μm.

We next examined whether blocking endocytosis could improve the survival rate of oocytes from *Tsg101* ^*d/d*^ mice by preventing the formation of defective endosomes. At 4°C, general endocytosis is blocked^30^ and oocytes are arrested at the GV stage owing to microtubule depolymerization at this temperature.^31^ As expected, all oocytes cultured at 4°C (Figure 4A) were arrested at the GV stage. Formation of EEA1‐positive puncta decreased in both groups after 5 h at 4°C (Figure 4B). At 8–10 h, oocytes from *Tsg101* ^*d/d*^ mice at 4°C exhibited a decrease in ooplasmic shrinkage from 28% to 0% (Figure 4C, at 8–10 h). By the end of culture at 4°C, 28% and 63% of oocytes from *Tsg101* ^*f/f*^ and *Tsg101* ^*d/d*^ mice, respectively, had degenerated (Figure 4C, at 17–24 h, Table S2). These results suggest that blocking endocytosis in oocytes of *Tsg101* ^*d/d*^ mice is effective in preventing cytoplasmic shrinkage during the initial stages.

Oocytes from Group II *Tsg101* ^*d/d*^ mice exhibited externally protruding blebs on their PM (Figure 2A). To examine if such bleb formation is suppressed at 4°C, we compared the status of PM in oocytes cultured at 37 or 4°C. For this comparison, we added IBMX, a blocker of nuclear envelope breakdown, to oocytes cultured at 37°C, to maintain the same nuclear configuration at the GV stage. During a 9‐h culture at 4°C, oocytes from *Tsg101* ^*d/d*^ mice mostly presented as healthy GVs, with 5% dead oocytes. At 37°C, approximately 60% of the oocytes showed cytoplasmic shrinkage (Figure 4D). The oocytes were subjected to CellMask staining to assess bleb formation. As shown in Figure 4E, 55% of oocytes from *Tsg101* ^*d/d*^ mice cultured at 37°C showed blebs, but not those cultured at 4°C. These results suggest that cytoplasmic shrinkage and bleb formation in *Tsg101*‐deficient oocytes are associated with cold‐suppressible cellular processes.

### 3.7 Deletion of *tsg‐101* in *C. elegans* increases membrane permeability of oocytes and embryos

**FIGURE 5:**
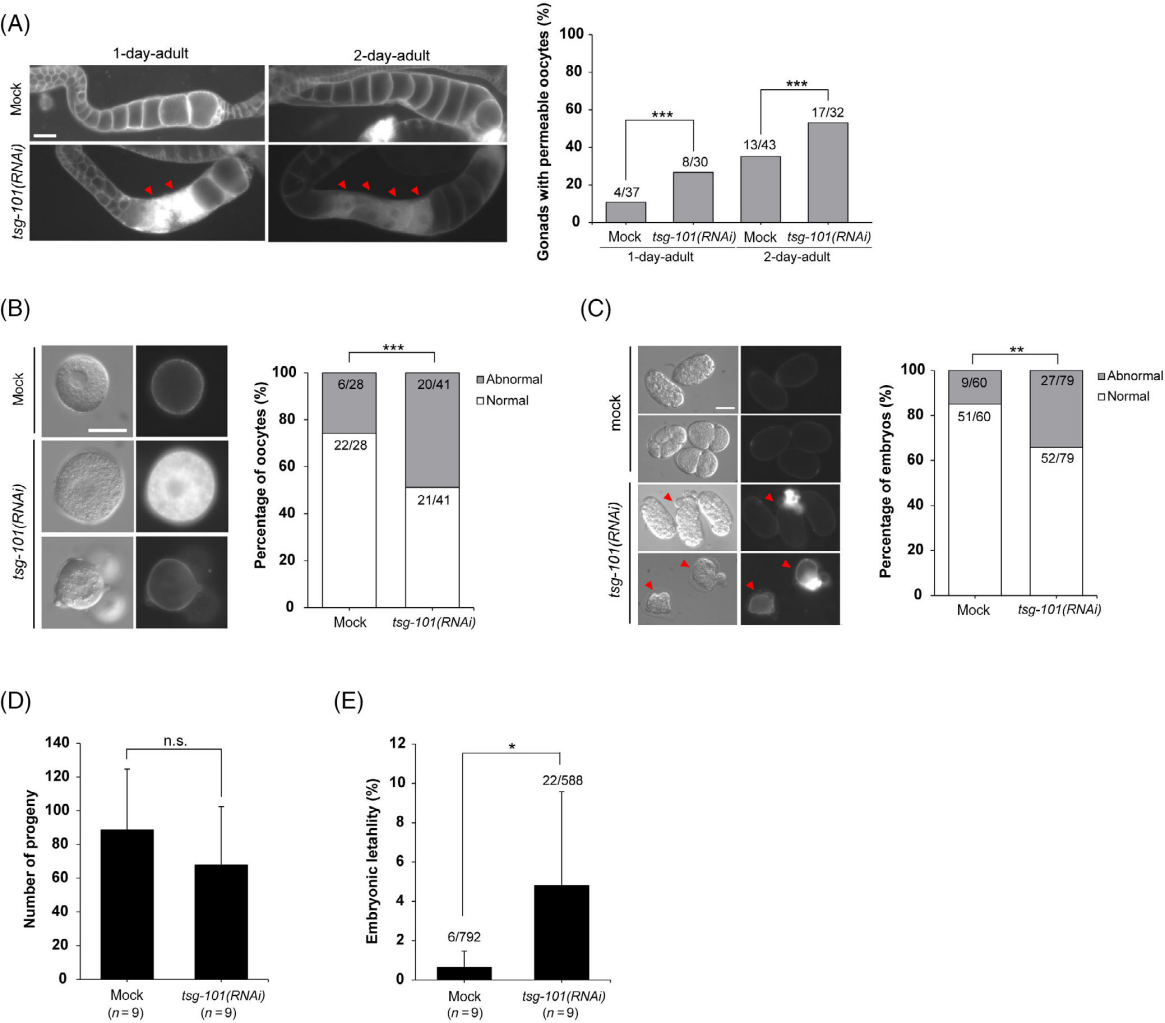
Depletion of *tsg‐101* increases the permeability of oocytes and embryos in *Caenorhabditis elegans*. (A) Oocyte membrane permeability was examined using lipophilic dye FM4‐64 in the extruded gonads of 1‐ and 2‐day adult hermaphrodites with mock and *tsg‐101* RNAi in the germ line. The graph shows the percentage of gonads containing permeable oocytes. Arrowheads indicate oocytes permeable to FM4‐64. Scale bar = 20 μm. ***p < 0.001 (chi‐square test). (B) Oocyte morphology of 2‐day adult hermaphrodites was analyzed after mock and germline t*sg‐101* RNAi. The graph shows the percentage of oocytes classified based on their morphological traits. The morphology of normal oocytes was circular and non‐permeable to FM4‐64. Abnormal oocytes are blebbed and/or permeable to FM4‐64. Scale bar = 20 μm. **p < 0.001 (chi‐square test). (C) Embryo morphology of 2‐day adult hermaphrodites was analyzed after mock and germline *tsg‐101* RNAi treatments. The graph shows the percentage of embryos classified according to their morphological traits. The morphology of normal embryos is ovoid and non‐permeable to FM4‐64, whereas abnormal embryos are shrunken and/or permeable to FM4‐64. Arrowheads indicate abnormal embryos. Scale bar = 20 μm. ***p < 0.001 (chi‐square test). (D) The number of progenies produced by 2‐day adult hermaphrodites after 24 h was counted after treatment with mock and germline *tsg‐101* RNAi. n.s., not significant (unpaired two‐tailed *t*‐test). (E) The percentage of dead embryos among the total number of progenies was measured after treatment with mock and germline *tsg‐101* RNAi. *p < 0.05 (unpaired two‐tailed *t*‐test).

Four components of the ESCRT I complex have been characterized in *C. elegans* and have been shown to be involved in receptor downregulation and abscission,^32^ similar to their mammalian counterparts. The observation that membranous structures and PM in *Tsg101*‐deficient oocytes are widely defective prompted us to examine whether there is a genetically conserved role in this aspect in *C. elegans* oocytes and embryos. L4‐stage worms were soaked in *tsg101*‐dsRNA for 24 h (1‐day adult) or 48 h (2‐day adult). Their gonads were examined for morphological defects and membrane permeability in oocytes and embryos. As shown in Figure 5A, the number of gonads with permeable oocytes was significantly higher in the *tsg‐101* RNAi‐treated worms. Approximately 50% of oocytes from the *tsg101*‐RNAi group showed PM blebbing and were permeable to FM4‐64 dye, suggesting membrane defects (Figure 5B). The embryos of *tsg101*‐RNAi‐treated 2‐day adults also showed similar abnormalities (Figure 5C). Whereas the number of progenies was not significantly different between the mock‐ and *tsg101* RNAi‐treated groups, the percentage of lethal embryos was much higher in the latter (Figure 5D,E). Thus, similar to mouse oocytes, *tsg‐101* appears to be required to maintain the PM integrity in *C. elegans* oocytes.

## 4 DISCUSSION

At the organismal level, *Tsg101* is indispensable for survival from early developmental stages.^9^, ^10^ In cell systems, *Tsg101* deletion leads to cell cycle defects, abnormalities in endolysosomal structures, and the accumulation of autophagic vacuoles, eventually leading to cell death.^10^, ^11^, ^33^, ^34^, ^35^ As shown herein, *Tsg101* deletion in mouse oocytes produced a unique age‐dependent effect. Oocytes from prepubertal *Tsg101* ^*d/d*^ mice are normally mature, fertilized, and developed to the blastocyst stage, whereas oocytes from peripubertal *Tsg101* ^*d/d*^ mice exhibit batteries of defects such as cytoplasmic shrinkage, PM rupture, and death. These death phenotypes, accompanied by compromized maturation, endolysosomal abnormalities, and PM leakage, developed after 28 days of age and rendered *Tsg101* ^*d/d*^ female mice completely infertile (no viable pups after months of mating, data not shown). We show herein that Tsg101 seems primary to function in the maturation of the endolysosomal pathway which dynamically develops in oocytes. Endosome formation and maturation are impaired in *Tsg101*‐deficient oocytes and blockade of this process at 4°C relieves them from cytoplasmic shrinkage and bleb formation. Blocking of endocytosis provides only temporary relief from cytoplasmic shrinkage and death ensues. It is surmized that the integrity of PM or other endomembranous structures is compromized in the absence of *Tsg101* in oocytes. This notion is further supported by the results of *tsg‐101* deletion in *C. elegans* oocytes and embryos, where its deletion increased the membrane permeability (Figure 5).

A mixture of healthy and degenerating oocytes was observed in the ovaries of 12‐week‐old *Tsg101* ^*d/d*^ mice (Figure S4). Oocyte demise in *Tsg101* ^*d/d*^ mice seems to be associated with changes in sexual maturation and follicular development. When the mouse reaches sexual maturation, a process requiring Tsg101 begins in oocytes, and the outcome of *Tsg101* deficiency is only visible when they progress through the maturation process. However, the mechanism underlying the temporal transition of the phenotypes in *Tsg101*‐deficient oocytes remains unclear. Herein, we highlight three cellular and physiological changes that may form the basis of this transition. First, the patterns of the EEA1‐positive structures change during this transition. In 15‐day‐old mice, EEA1‐positive signals in oocytes showed irregular shapes, unlike the small puncta pattern observed in various cell types.^36^ Such an irregular pattern may indicate that early endosomes are yet to be functionally organized. As mice ages, EEA1‐positive puncta became a typical, small, and round pattern in oocytes (Figure 3A). This dynamic change in the EEA1 pattern may be associated with the age‐dependent phenotype of *Tsg101*‐deficient oocytes. Up to 4 weeks of age, the diameter of oocytes increases to an average of 80 μm.^37^ When oocytes are still growing and ready for PB extrusion, a larger PM area may need to be preserved. Thus, oocytes from mice younger than 4 weeks of age may restrict endocytosis, as endocytic activity decreases the surface area of PM by inward folding and internalization.^38^ Thus, a low rate of endocytosis in the oocytes of young mice may be necessary to retain the surface area of the PM for further growth up to a certain age. When endocytic activity increases with age, the absence of Tsg101 may be detrimental to oocyte survival. However, this hypothesis requires further investigation.

Second, a change in the physiological environment during puberty may influence the death of Tsg101‐deficient oocytes. In mammals, the activation of the hypothalamic‐pituitary‐gonadal axis drives sexual maturation; vaginal opening in female mice is first observed at approximately 4 weeks of age, whereas the first natural ovulation generally begins at approximately 5–6 weeks of age.^39^, ^40^ GnRH, the initial conductor of the hypothalamic‐pituitary‐gonadal axis, induces follicle‐stimulating hormone secretion, which causes follicular growth. During folliculogenesis, signaling crosstalk between oocytes and surrounding somatic cells is actively ongoing to prepare oocytes for ovulation.^4^, ^41^ There is evidence that TNFα increases in ovarian lysates during the first ovulation at puberty in rodents.^42^ Changes in the expression of such signaling mediators may cause the differential response to *Tsg101* deletion.

Third, a change in the physical characteristics of the oolemma may be associated with oocyte death at a specific time. Mammalian oocytes sized around 80‐100 μm in diameter, exhibit the lowest surface‐to‐volume ratio among all animal cells. The mechanical properties of the oolemma change during maturation showing and rapid softening after germinal vesicle breakdown (GVBD) and loss of membrane or cortical tension.^43^, ^44^ From the GVBD to MI stage, which is approximately 2–7 h from the beginning of *in vitro* maturation, oocyte stiffness decreases dramatically.^44^ The molecular nature of this change is yet to be established, but the timing of the membrane change is coincident with membrane rupture in oocytes from Group II *Tsg101* ^*d/d*^ mice (Movie S4). Cellular defects in oocytes from Group II *Tsg101* ^*d/d*^ mice are associated with other phospholipid‐bound structures, such as endosomes and lysosomes. Thus, the age‐dependent death phenotype in oocytes from *Tsg101* ^*d/d*^ mice also indicates temporal changes in the integrity of these membranous structures. Along with the results of our previous study showing a change in phospholipid profiles between oocytes from young and aged mice,^28^ Tsg101 may also be involved in the maintenance of physical integrity during the remodeling of membranous structures.

The puncta‐like localization of microinjected EGFP‐Tsg101 (Figure 2D), similar to the pattern observed in other cell systems,^6^ indicates a role in endolysosomal maturation. Peripheral localization of EGFP‐Tsg101 in dying oocytes (Figure S5B) may also indicate a role of Tsg101 in the response to PM damage (Figure S5B). This pattern has been observed in cells with intentionally caused damage.^15^ Large membrane blebs were observed in oocytes from Group II *Tsg101* ^*d/d*^ mice (Figure 2A) and abrogated at 4°C (Figure 4E). Such blebbing may be associated with endocytic events on PM and may be due to the malfunction of membrane scission machinery in the absence of Tsg101, Tsg101, and other ESCRT factors that are implicated in membrane scission away from the cytosol.^45^

In human oocytes undergoing *in vitro* fertilization, the presence of membranous granules and blebs within the perivitelline space is associated with decreased implantation and pregnancy outcomes.^46^ Ovarian hyperstimulation protocols for *in vitro* fertilization routinely use GnRH agonists or antagonists, which may affect oocyte viability under compromized ESCRT function, as shown here. Whether defective or decreased efficiency of ESCRT machinery in oocytes is one cause of poor developmental competence after *in vitro* fertilization remains an unexplored area. Investigations along this path will contribute to the understanding of the mechanisms involved in reproductive aging with the potential to alleviate infertility.

## AUTHOR CONTRIBUTIONS

Hyejin Shin and Hyunjung Jade Lim devised the study; Hyejin Shin, Dayoung Park, Jiyeon Kim, Min‐Yeong Nam, Kwon Sojung, Da‐Eun Um, Ji‐Eun Oh, Esther Youn, Jin Hyun Jun, and Hye‐Ryun Kim performed the experiments; Hyejin Shin, Dayoung Park, Jiyeon Kim, Min‐Yeong Nam, Kwon Sojung, Da‐Eun Um, Ji‐Eun Oh, Yhong‐Hee Shim, Jin Hyun Jun, Haengseok Song, and Hyunjung Jade Lim analyzed the data; Kay‐Uwe Wagner provided the materials; Hyejin Shin, Dayoung Park, Jiyeon Kim, Min‐Yeong Nam, Yhong‐Hee Shim, and Hyunjung Jade Lim wrote the manuscript with input from all authors.

## FUNDING INFORMATION

This work was supported by a National Research Foundation of Korea (NRF) grant (NRF‐2020R1A2C1004122) funded by the Korean government (MSIT), by the Basic Science Research Program (NRF‐2018R1D1A1B07045205) through the National Research Foundation of Korea (NRF) funded by the Ministry of Education. The maintenance of *Tsg101* mutant mice was supported, in part, by the Public Health Service grant CA219332 (to Kay‐Uwe Wagner). The funders had no role in the study design, data collection, analysis, decision to publish, or manuscript preparation.

## CONFLICT OF INTEREST

The authors declare that they have no competing interests.

## Supporting information


**Appendix S1** Supporting information.

**Movie S1.** *In vitro* maturation of Group I *Tsg101* ^*f/f*^ oocytes. Photos were automatically taken at 1‐h intervals using a JuLI^TM^ time‐lapse microscope (Digital Bio, JuLI‐b004) and compiled.

**Movie S2.** *In vitro* maturation of Group I *Tsg101* ^*d/d*^ oocytes.

**Movie S3.** *In vitro* maturation of Group II *Tsg101* ^*f/f*^ oocytes.

**Movie S4.** *In vitro* maturation of Group II *Tsg101* ^*d/d*^ oocytes.

## Data Availability

The data that support the findings of this study are available from the corresponding author upon reasonable request.
