# Supplementary material for: Peripubertal requirement of Tsg101 in maintaining the integrity of membranous structures in mouse oocytes
Source: Cell Prolif. 2022 Jun 29;55(10):e13288. doi: 10.1111/cpr.13288 (PMC9528763; doi:10.1111/cpr.13288)
Supplement: Supplementary file 1 — Appendix S1 Supporting information. [file CPR-55-e13288-s004.docx]

Supplementary Information for:

**Peripubertal requirement of Tsg101 in maintaining the integrity of membranous structures in mouse oocytes**

Shin et al.

**This file includes:**

Figures S1 to S6

Table S1 and S2

Captions for Movies S1 to S4

**Other supplementary materials for this manuscript include the following:**

Movies S1 to S4

**
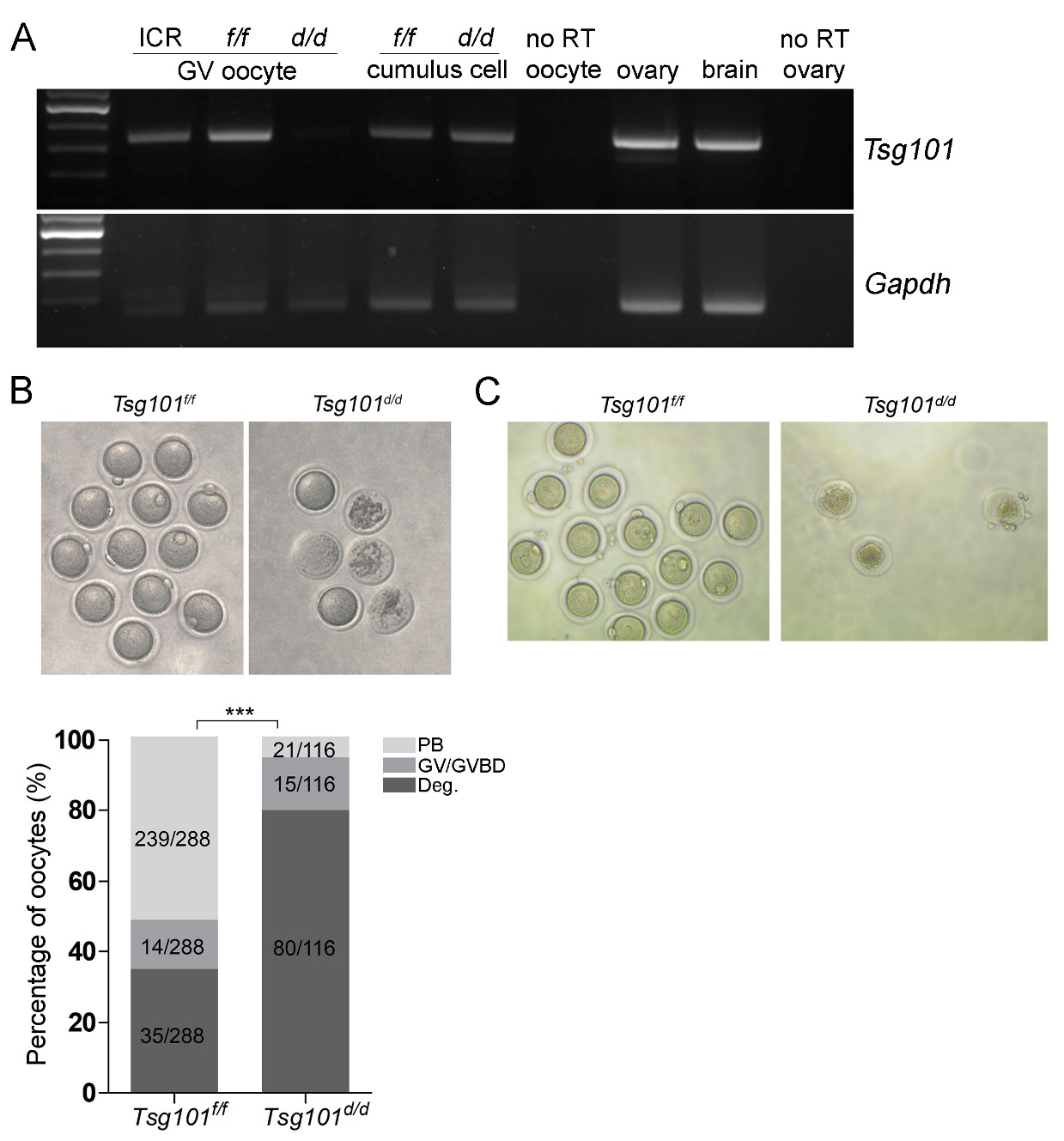
**

**Figure S1. Oocyte demise in *Tsg101^d/d^* mice.**

(A) Deletion of *Tsg101* in oocytes was confirmed by RT-PCR. GV oocytes (n = 100) from 4-week-old mice (n=7) were pooled for RNA preparation. Cumulus cells removed from the GV oocytes were pooled and used for RNA preparation. RNAs from mouse ovary and brain were used as positive controls. *GAPDH* was used as an internal loading control. Similar experiments were performed twice with different sets of samples and produced similar results. RT, reverse transcription; no RT, no reverse transcription. Primer sequences are listed in Supplementary Table 1. (B) Group II *Tsg101^f/f^* (n = 16) and *Tsg101^d/d^* (n = 17) mice received PMSG and hCG treatment, respectively, and ovulated PB oocytes were collected from the oviducts at 13 h post-hCG treatment. The rates of *in vivo* maturation in *Tsg101^f/f^* and *Tsg101^d/d^* mice are shown in the graph. The number of oocytes per total number of oocytes is shown in each bar. GV, germinal vesicle; GVBD, GV breakdown; PB, polar body; Deg., degenerated. (C) Set of representative images showing ovulated oocytes from Group III *Tsg101^f/f^* and *Tsg101^d/d^* mice.

**
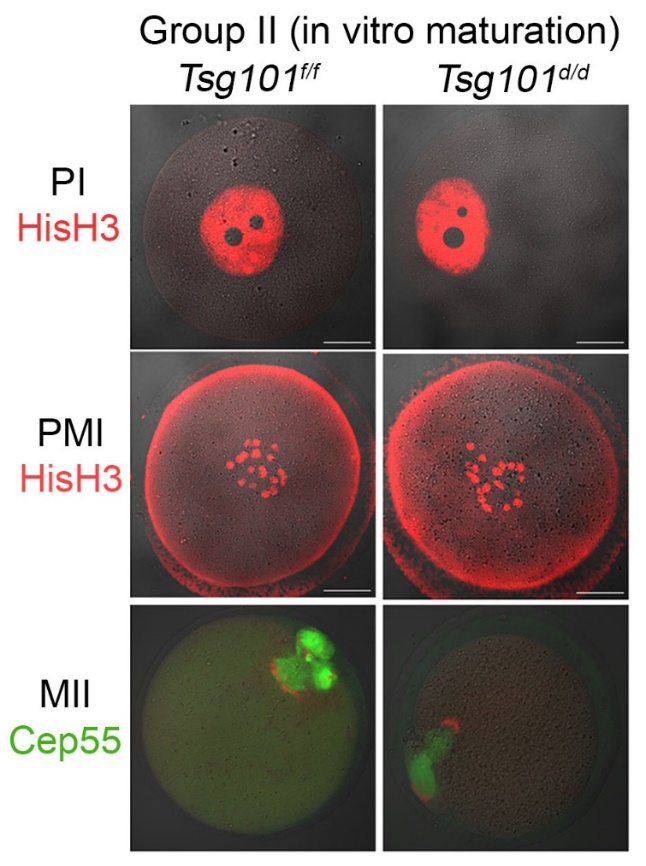
**

**Figure S2. Immunofluorescence staining of histone H3 and Cep55 in oocytes from Group II *Tsg101^f/f^* and *Tsg101^d/d^* mice.**

GV oocytes matured *in vitro*. At 3 h (prometaphase I, PMI) and 12 h (metaphase II, MII), surviving oocytes were subjected to immunofluorescence staining as indicated. Normal localization of histone H3 (HisH3) in the nuclei (PI and PMI, red) and of Cep55 on the spindle and midbody (MII, green) were observed in both *Tsg101^f/f^* and *Tsg101^d/d^* oocytes. Scale bar = 20 μm.


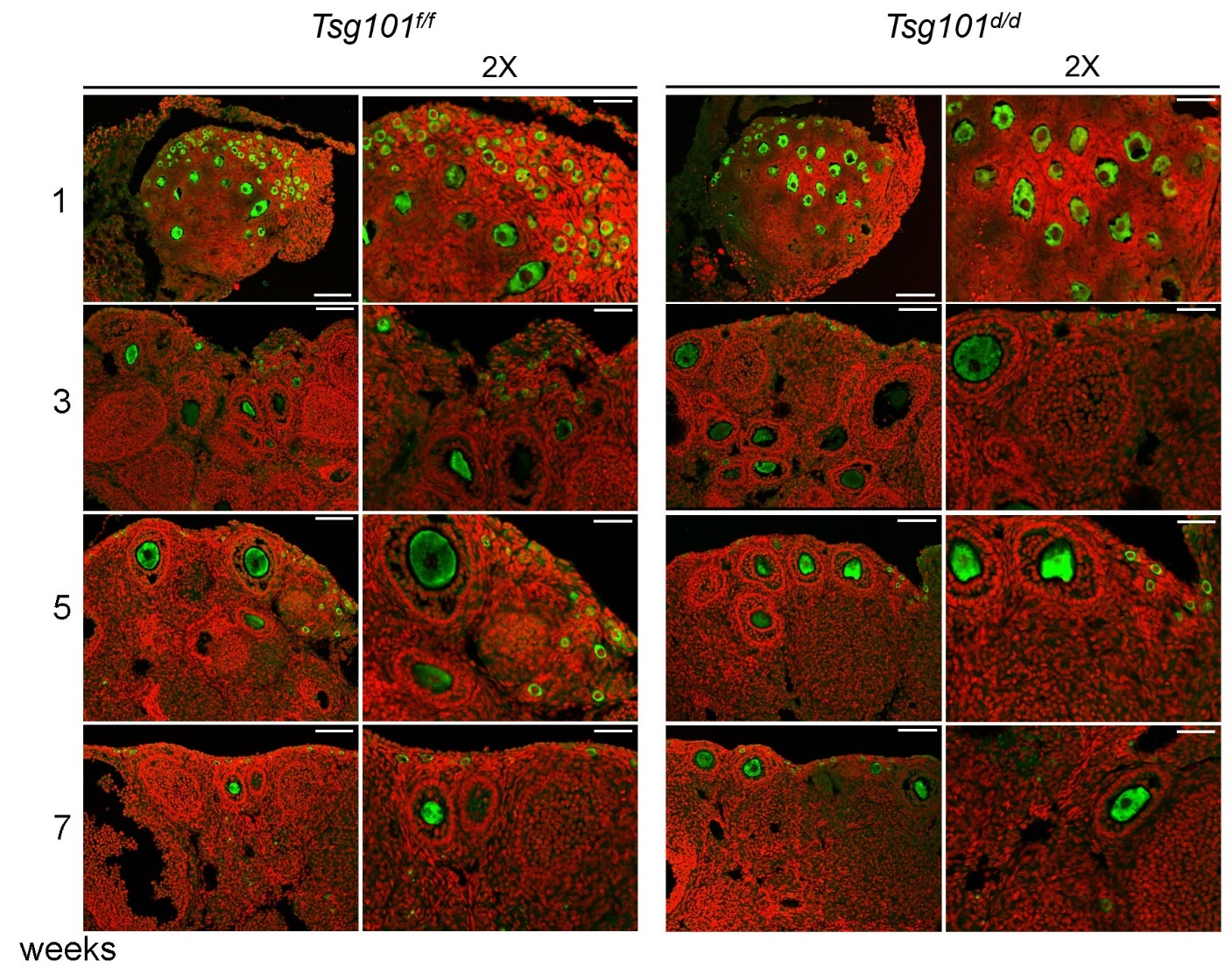


**Figure S3. Immunofluorescence staining of MVH in the ovaries of *Tsg101^f/f^* and *Tsg101^d/d^* mice.**

Cryosections of the collected ovaries were subjected to immunofluorescence staining with an anti-MVH antibody (a germ cell marker) at the indicated ages (weeks). Cryosections were fixed with 4% paraformaldehyde, treated with an anti-MVH antibody, and probed with an Alexa Fluor 488-conjugated secondary antibody (green). The primordial germ cell pool near the ovarian surface was normally observed in all age groups of *Tgs101^d/d^* mice. More than two mice were used in independent experiments. Scale bar = 100 μm.


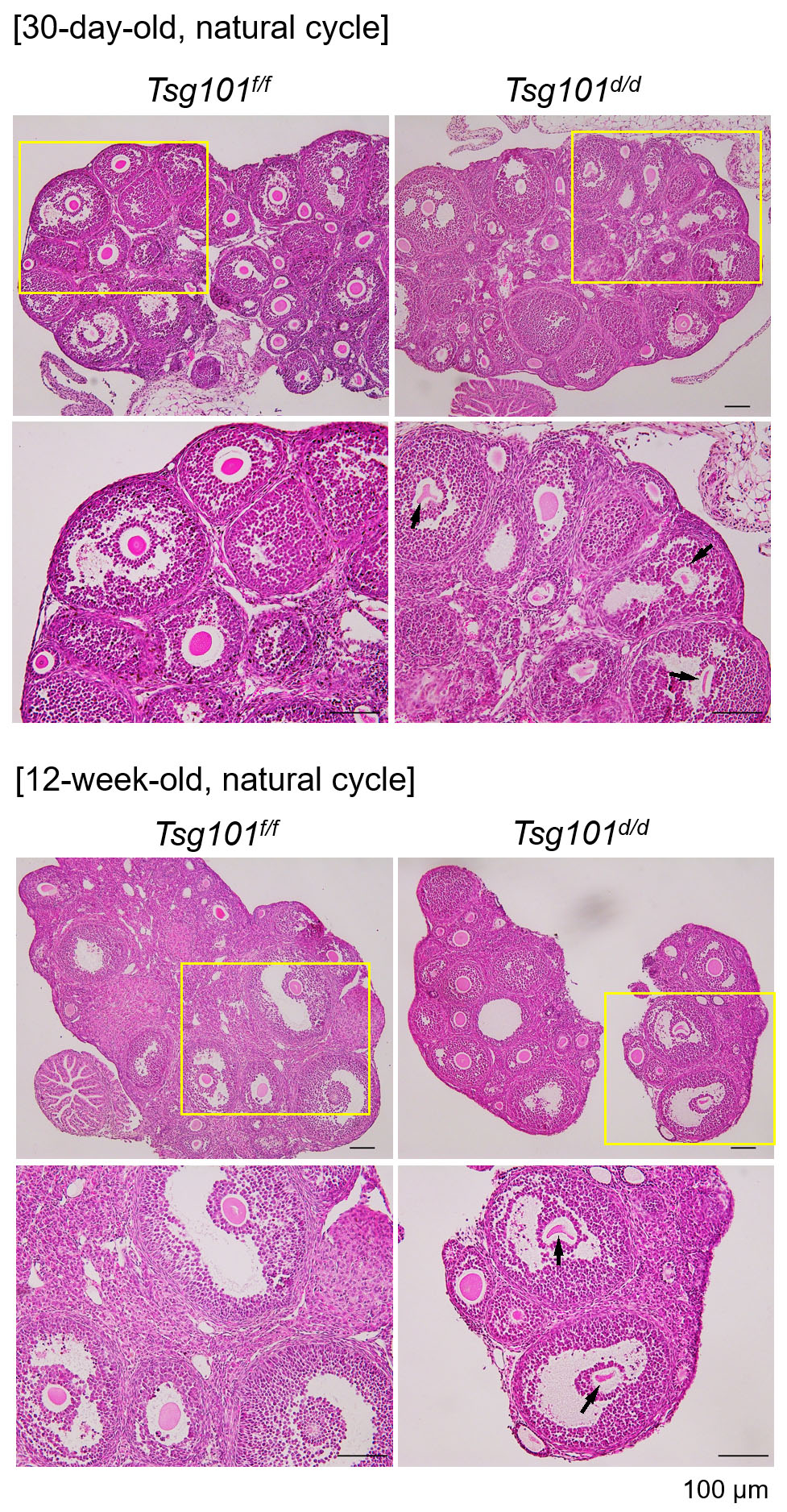


**Figure S4. Histological analyses of ovaries from naturally cycling *Tsg101^f/f^* and *Tsg101^d/d^* mice at indicated ages.**

Paraffin-embedded sections (6-μm thickness) were stained with hematoxylin and eosin. The mice naturally cycled up to the indicated ages. The yellow rectangles are magnified in the panel below. Arrows indicate unhealthy degenerating oocytes within the large follicles of *Tsg101^d/d^* mice at both 30 days and 12 weeks of age. Oocytes within smaller follicles appear normal.

**
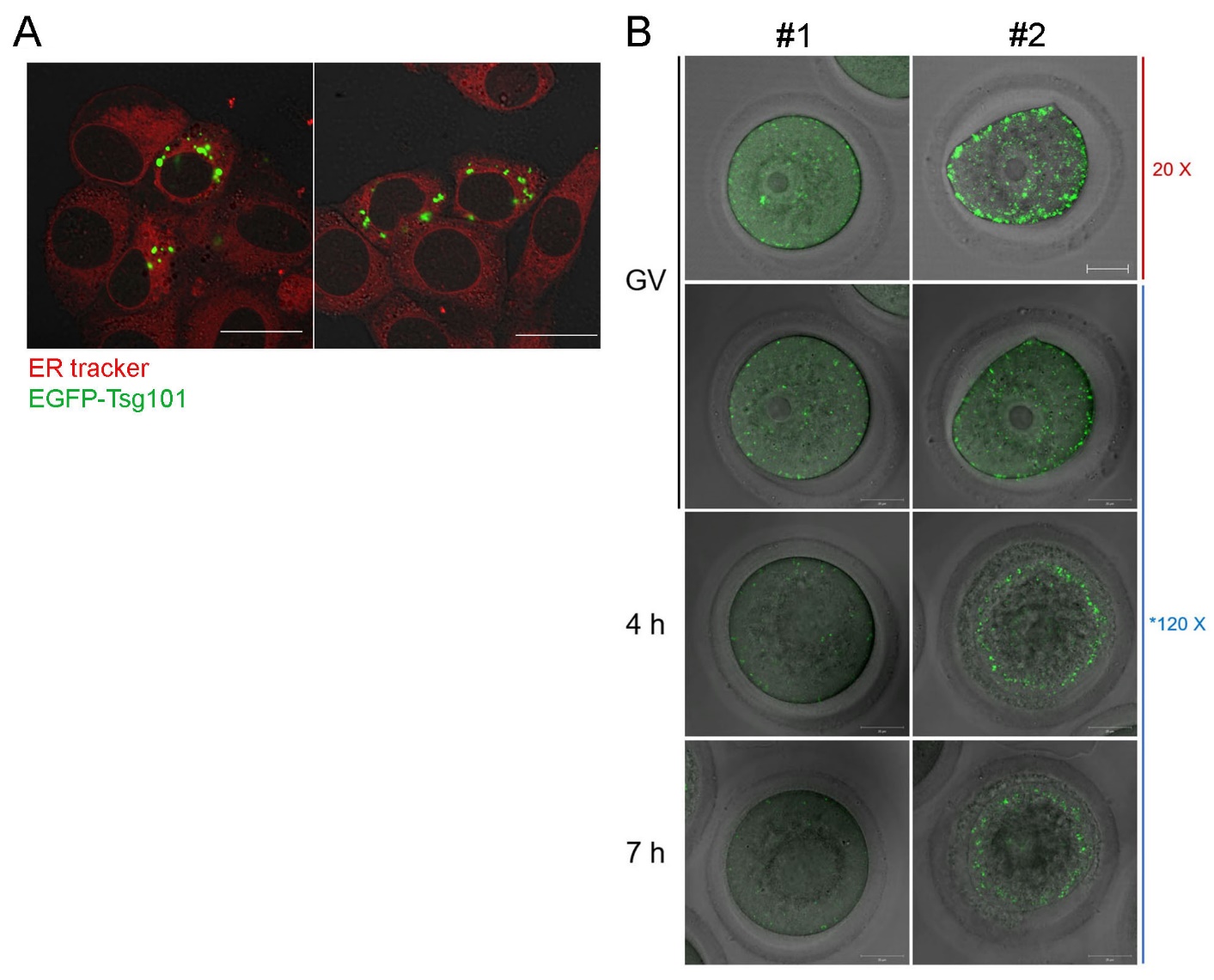
**

**Figure S5. Localization of EGFP-Tsg101 in oocytes after microinjection.**

(A) NIH3T3 cells were transfected with the plasmid, and expression was examined by confocal live imaging at 48 h later. As reported previously in studies using transfected cell lines, the EGFP-Tsg101 construct exhibited puncta-like patterns in the cytoplasm. Cells were counterstained with ER-Tracker Red. This construct was used to produce *EGFP-Tsg101* cRNA for oocyte microinjection (Fig. 2d). Scale bar = 20 μm. (B) Two EGFP-Tsg101 RNA-microinjected oocytes showing different localization. The pattern of #1 oocyte is typical (Fig. 2d). We observed a degenerating oocyte (#2) showing peripheral accumulation of EGFP-Tsg101 signal. At 20X, such accumulation is highly visible (top, right panel). Oocyte #2 degenerated during maturation, with EGFP-Tsg101 signal decorating the PM.


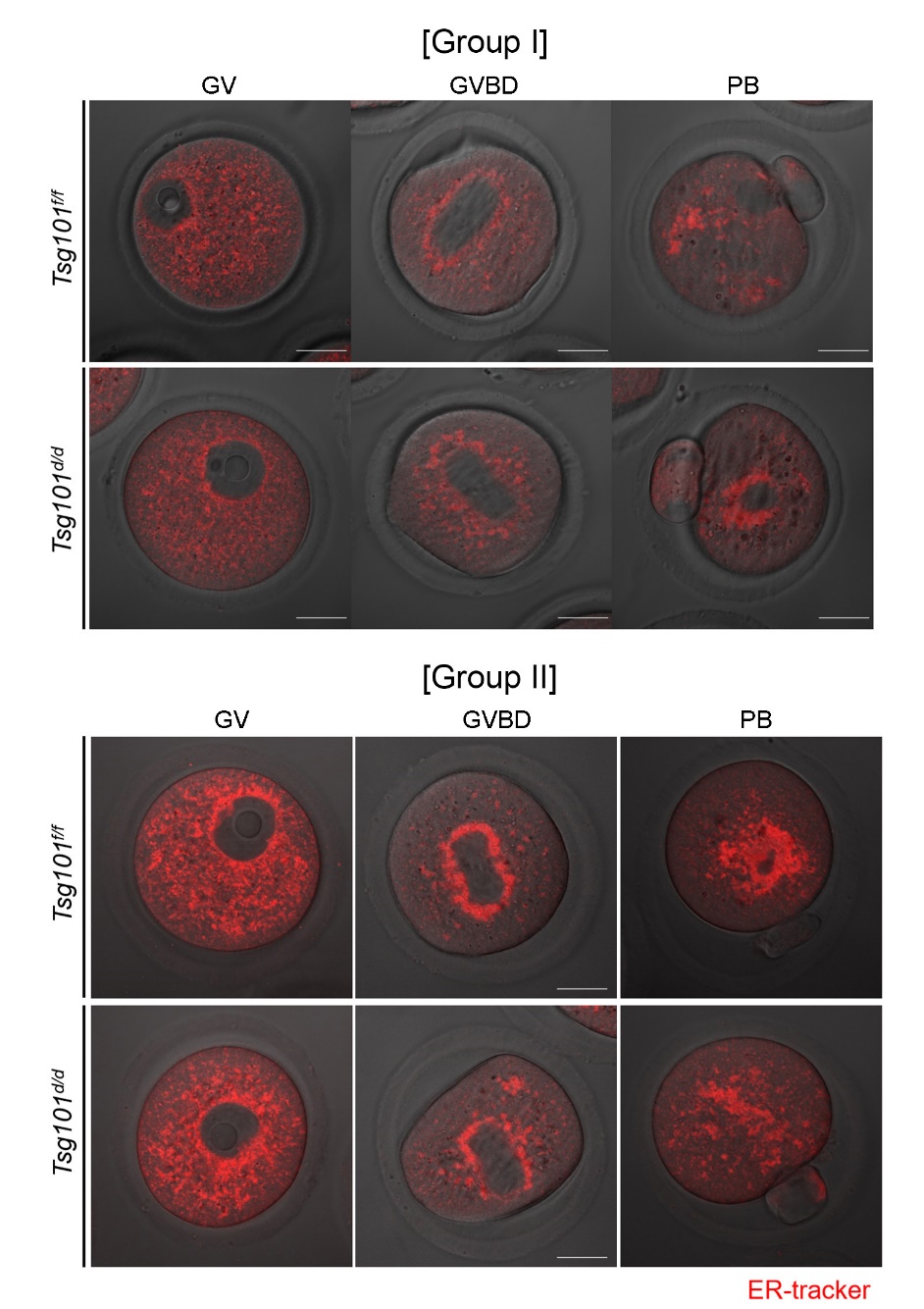


**Figure S6.** Distribution of the endoplasmic reticulum in oocytes from *Tsg101^f/f^* and *Tsg101^d/d^* mice. Oocytes were stained with ER-Tracker Red and observed by confocal live imaging. For Group I, the experiment was performed once using three mice in each group. For Group II, the experiment was performed twice using 3 *Tsg101^f/f^* and 5 *Tsg101^d/d^* mice. Scale bar = 20 µm.

**Table S1. Primers used for PCR analyses.**

| **Gene** | **Sequence (5′-3′)** | **Size**  **(bp)** | **GenBank accession no.** | **Use** |
| --- | --- | --- | --- | --- |
| *Tsg101* wild | F: ACA CAT ACC CAT ATA ACC C  R: CTC CCA ACC CCA GTG GTT AT | 332 | NM_021884.3 | genotyping |
| *Tsg101* floxed | F: ATG GCG GTG TCC GAG AGT CAG  R: CAC GGT CAG AGT TGC TGG AG | 193 | NM_021884.3 | genotyping |
| *Zp3*-*cre* positive | F: CTA GGC CAC AGA ATT GAA AGA TCT  R: GTA GGT GGA AAT TCT AGC ATC ATC C | 324 | NM_000071.6 | genotyping |
| *Zp3*-*cre* transgene | F: GCG GTC TGG CAG TAA AAA CTA TC  R: GTG AAA CAG CAT TGC TGT CAC TT | 100 | NM_000071.6 | genotyping |
| *Tsg101* | F: ACA CAT ACC CAT ATA ACC C  R: CTC CCA ACC CCA GTG GTT AT | 332 | NM_021884.4 | RT-PCR  (Figure S1) |
| *Gapdh* | F: TGC CCC CAT GTT TGT GAT G  R: CAC GGT CAG AGT TGC TGG AG | 151 | NM_001289726.1 | RT-PCR  (Figure S1) |
| *Tsg101* | F: ATG GCG GTG TCC GAG AGT CAG  R: TTG ACA GTT TGA CGG ACG GT | 80 | NM_021884.4 | qPCR  (Figure 2E) |
| *Chmp4b* | F: GGA GAA GAG TTC GAC GAG GAT  R: TGG TAG AGG GAC TGT TTC GGG | 111 | NM_029362.3 | qPCR  (Figure 2E) |
| *H2afz* | F: ACA GCG CAG CCA TCC TGG AGT A  R: TTC CCG ATC AGC GAT TTG TGG A | 202 | NM_016750.3 | qPCR  (Figure 2E) |

F, Forward; R, Reverse; qPCR, quantitative PCR

| Genotype | No. of  mice | Total  GV | Temp. | Time | GV (%) | GVBD (%) | PB  (%) | Shrunken (%) | Deg.  (%) |
| --- | --- | --- | --- | --- | --- | --- | --- | --- | --- |
| *Tsg101^f/f^* | 14 | 68 | 37 °C | 5 h | - | 68 | - | - | - |
|  |  |  |  | 8 -10 h | - | 68 | - | - | - |
|  |  |  |  | 17 -24 h | - | - | 68 | - | - |
|  |  | 67 | 4 °C | 5 h | 66 | - | - | - | 1 |
|  |  |  |  | 8 -10 h | 66 | - | - | - | 1 |
|  |  |  |  | 17 -24 h | 48 | - | - | - | 19 |
| *Tsg101^d/d^* | 21 | 72 | 37 °C | 5 h | 20 | 45 | - | 5 | 2 |
|  |  |  |  | 8 -10 h | 15 | 30 | 2 | 20 | 5 |
|  |  |  |  | 17 -24 h | 4 | 1 | 25 | 24 | 18 |
|  |  | 72 | 4 °C | 5 h | 7 | - | - | - | 1 |
|  |  |  |  | 8 -10 h | 71 | - | - | - | 1 |
|  |  |  |  | 17 -24 h | 27 | - | - | - | 45 |

**Table S2. *In vitro* maturation of oocytes from *Tsg101^f/f^* and *Tsg101^d/d^* mice at 37 °C or 4 °C (Fig. 4a).**

Movie S1. *In vitro* maturation of Group I *Tsg101^f/f^* oocytes. Photos were automatically taken at 1-h intervals using a JuLI^TM^ time-lapse microscope (Digital Bio, JuLI-b004) and compiled.

Movie S2. *In vitro* maturation of Group I *Tsg101^d/d^* oocytes.

Movie S3. *In vitro* maturation of Group II *Tsg101^f/f^* oocytes.

Movie S4. *In vitro* maturation of Group II *Tsg101^d/d^* oocytes.
